# Can workplace intervention prolong work life of older workers? A quasi-experimental study

**DOI:** 10.1007/s00420-022-01919-8

**Published:** 2022-09-06

**Authors:** Subas Neupane, Saila Kyrönlahti, Jodi Oakman, Anna Siukola, Aart-Jan Riekhoff, Susan Kuivalainen, Clas-Håkan Nygård

**Affiliations:** 1 Unit of Health Sciences, Faculty of Social Sciences, Tampere University, Arvo Ylpönkatu 34, 33520 Tampere, Finland; 2 Gerontology Research Center, Tampere University, 33014 Tampere, Finland; 3 Centre for Ergonomics and Human Factors, School of Psychology and Public Health, La Trobe University, Melbourne, VIC 3086 Australia; 4 Clinical Medicine, Faculty of Medicine, Health and Technology, Tampere University, Arvo Ylpönkatu 34, 33520 Tampere, Finland; 5 Finnish Centre for Pensions, Elaketurvakeskus, 00065 Helsinki, Finland

**Keywords:** Workplace intervention, Aging workers, Prolonged work life, Disability pension

## Abstract

**Background:**

We aimed to evaluate the impact of a workplace senior program intervention on early exit from labor market and on the disability retirement among older employees and work-related physical factors associated with it.

**Methods:**

A total of 259 individuals aged 55 + years participated in the study (107 in intervention and 152 were controls). A questionnaire survey was conducted among Finnish food industry employees between 2003 and 2009 and the intervention “senior program” was provided between 2004 and 2009. The type of pension for the respondents who had retired by 2019 was obtained and dichotomized as statutory vs. early labor market exit. Disability pension was investigated as a separate outcome. Information on work-related factors was obtained from the survey. Cox regression analysis was used to estimate hazard ratios (HR) with their 95% confidence intervals.

**Results:**

Fifty-one employees had early labor market exit. Of them, 70% (*n* = 36) were control participants. Employees in the senior program worked for longer years (mean years 7.4, 95% CI 6.4–8.1) compared to the control (6.6, 95% CI 6.3–7.5). Sixty percent lower risk of early labor market exit (HR 0.40, 95% CI 0.19–0.84) and disability pension was found among employees in the senior program compared to the control group. Good work ability had a 94% lower risk (0.06, 95% CI 0.01–0.29) of early labor market exit and 85% lower risk (0.15, 95% CI 0.03–0.73) of disability pension compared to poor work ability. Employees with musculoskeletal pain had 4 times higher risk of disability pension compared to those without musculoskeletal pain.

**Conclusions:**

A workplace senior program intervention prolonged work life and had positive effect on reducing disability pension among older industrial workers.

**Supplementary Information:**

The online version contains supplementary material available at 10.1007/s00420-022-01919-8.

## Introduction

The global population is aging, resulting in a decreasing labor market and pressure on pension schemes (Kühn et al. 2018). Sustainable employment is critical to ensure that workers can remain at work to meet the needs of the labor market. For workers to remain employed, individuals need to be able to work longer, and the working conditions need to be appropriate for older workers to meet their capacities. Workplace interventions to support older workers in staying at work are critical, but lack evidence to determine what is required and then measure their effectiveness.

Retirement from work is influenced by a range of push and pull factors, which include personal characteristics, work ability, and job satisfaction, along with the physical and psychosocial workplace environment (Shacklock et al. 2009; Oakman and Wells 2013; Neupane et al. 2022). A particular challenge arises with physically demanding work for older workers who are more likely to have chronic conditions such as musculoskeletal diseases due to prolonged exposures and are commonly associated with early retirement (Lahelma et al. 2012; Rijn et al. 2014). The role of heavy physical work with awkward postures (Gustafsson et al. 2019) and work with adverse psychosocial factors such as high job demands and poor social support (Leineweber et al. 2019) have been identified as increasing the risk of accessing a disability pension.

Work ability is a strong determinant of disability pension (Roelen et al. 2014; Jääskeläinen et al. 2016; Alavinia et al. 2009). Work ability is determined by the balance of individual’s work demands with the resources, which take into account the subjective experiences of a person’s ability to cope with physical and mental requirements at work with information on diseases and resulting functional limitation (Ropponen et al. 2020; Zwart et al. 2002). This concept was developed to identify the risks and promote work ability by tailoring interventions at the individual level (Ilmarinen and Tuomi 2004). Association of work ability with disability pension has been extensively reported earlier (Roelen et al. 2014; Jääskeläinen et al. 2016; Alavinia et al. 2009).

Interventions to improve working conditions and person environment fit may be an effective strategy to improve retention of older workers who are at risk of early exit from labor market or work-related disability. De Boer et al. (2007) reported a small effect of an education counseling intervention on improved work ability among Dutch workers, yet the intervention did not reduce work disability. A Norwegian study, which facilitated a work intervention among employees with reduced work capacity, resulted in reduced uptake of the disability pension (Midtsundstad and Nielsen 2016). Another study found that occupational health intervention programs were effective in preventing early retirement in the Netherlands (Boer et al. 2004). A Cochrane systematic review reported no convincing conclusion on the effectiveness of workplace interventions on preventing work disability (Oostrom et al. 2008). However, a more recent Cochrane review examined work disability in relation to sickness absence and found moderate-quality evidence of the effectiveness of interventions to reduce the duration of sickness absence (Vilsteren et al. 2015) which act as a precursor to accessing the disability pension (Leino-Arjas et al. 2021). Intervention studies which focus on prolonging work career and reducing disability retirement remain rare, but are needed to underpin evidence-based decisions to support the creation of working conditions to support a prolonged healthy and productive working life and extension of retirement age from employment for aging workers.

Interventions to prevent early exit from paid employment require an understanding on the determinants of disability pension access (Leino-Arjas et al. 2021; Albertsen et al. 2007; Krause et al. 1997). In an earlier study, we examined work disability, which may result from exposure to adverse working conditions early in an individual’s working life from sickness absence that had led to the premature disability (Leino-Arjas et al. 2021). Therefore, the complex nature of retirement and the subsequent access to pension schemes require longitudinal analysis of working conditions, and the relationship between these and access to pension schemes with employees follow into retirement.

The current study examined the effect of a workplace intervention, initiated and financed by the employer, focused on reducing early exit from labor market and addressing the associated work-related physical factors. The second aim was to study the effect of intervention on disability retirement and the associated work-related factors. The workplace intervention was implemented in a Finnish food industry company in 2004. The average retirement age increased (from 58 years in 2004 to 61.6 years in 2013) and according to unpublished surveys, the work well-being increased in the company. In 2010, the company won a Finnish national prize for innovative practices in employment and social policy and was later awarded a “Best agers EU-project” for good practices in Finland to prolong working at different life stages (MoPAct 2015). Currently, almost all of those who participated in the study program have retired from paid employment. The current study was conducted to examine if the nationally and internationally awarded intervention program also reduced work disability and resulted in a prolonged work career compared to workers who did not participate. This innovative knowledge may provide insights for other similar work sectors to encourage undertaking similar interventions at workplaces if this kind of approach is identified as effective at providng sustainable employment and prolonging working lives.

## Methods

A survey of food industry employees in Finland was conducted in 2003. The company employed about 2000 employees, mostly blue collar (≈80%) and women (≈60%) with a mean age of all employees 41 ± 9.7 years (range 20–66 years) at baseline. A repeated survey was conducted among all employees of the company in spring 2005, 2007 and 2009 with a response rate ranging from 56 to 72% (Siukola 2013; Neupane 2012). The respondents provided written consent for linking the survey data with information on age, gender and occupational status from the personnel registers. Information on the type of pension received by those who retired by 31st December 2019 was obtained from the Finnish Center for Pension and then linked to survey data.

Individuals aged 55 + years who answered at least one survey from 2003 to 2009 and participated in the senior program, or were assigned as controls and had information on their pension data, were included in the current study (*n *= 259). This study follows a quasi-experimental design as participation to the senior program was voluntary, with no ability to randomize participants. The researcher did not have control over the treatment. For those employees who had participated in more than one questionnaire survey, only one questionnaire responses from the earliest survey were used in the analysis. In total, 107 employees who participated in the senior program and 152 controls matched by their age with the intervention group respondents were analyzed. The majority of study participants (> 95%) were blue-collar workers. The intervention was implemented in a real-life setting; therefore, the participants for example may have responded to the survey in 2003 and participated in the intervention in 2008 or at any time in between.

## Workplace intervention

An intervention called the “senior program” was implemented as a voluntary program from company initiative between 2004 and 2009 and later continued as a standard workplace health promotion program open to all employees. The senior program was included as one of the company’s development activities. The aim of the program was to maintain and promote work well-being and work ability among older (55 + year) employees. Workers involved in the voluntary senior program firstly had individual discussions with their supervisors, their occupational health service and human resource personnel to plan a tailored program for the worker. The workers in senior program were allocated less physically demanding tasks and offered the option to participate in rehabilitation and training programs if needed. The requirement to work a three-shift roster was removed, and participants were awarded a holiday bonus which could be taken as additional annual leave. This bonus included an additional 15 days of holidays and the option to apply for “alteration leave”, a Finnish scheme similar to taking sabbatical leave (Siukola 2013). In the first, all 55 years or older subjects who did not join the program were included in the control group. In the following years, there were fewer new controls, as then only those who were 55 years and who did not join the program were included. One-to-one matching was not done in the controls. The control group was working as usual. The control group received the intervention when the program was continued as a standard workplace health promotion after 2008.

This study was approved by the ethical committee of Pirkanmaa Hospital District and written consent to link the survey information to their register data was obtained from each participant.

## Measurement of variables

### Early exit from the labor market

The main outcome was early exit from labor market. We defined early exit from the labor market if the employee was retired and on support other than the statutory pension, which included a full-time disability pension, rehabilitation support, a part-time pension, partial disability pension or an unemployment pension. The majority (53%) of those who received other than statutory pension were on a full-time disability pension. Four employees were receiving rehabilitation support (1 from the senior program and 3 from the controls) and did not return to work and were consequently included in the early labor market exit category.

### Disability pension

Disability pension was a secondary outcome and defined as those who retired with a full-time disability pension vs all other types of pension. The other type of pension included a statutory pension, a rehabilitation support, a part-time pension, a partial disability pension or an unemployment pension.

### Work-related variables

Work ability was measured by using the first item of the work ability index (WAI) (Tuomi et al. 1997) “current work ability compared with the lifetime best” with a possible response score of 0 (absolutely incapable to work) to 10 (work ability at its best). In the current analysis, we created three categories as poor (0–7), moderate (8) and good (9–10) (Gould et al. 2008).

Perceived physical strain was measured using a rating of perceived exertion (RPE) with the question “How physically hard/ exhausting do you feel your job is on a normal work-day?” on a scale from 6 (not at all) to 20 (very much) (Borg 1970). Physical strain was dichotomized in the current analysis as low (6–13) and high (14–20) using the median value as a cutoff point.

Repetitive movements and awkward postures were measured as “Do movements/postures cause inconvenience or strain in your work?” and reply options on a five-point Likert scale (1 = not at all, 5 = very much) were given. The variables were dichotomized into low (1–2) and high (3–5) using the median value as the cutoff point.

Environmental exposure was measured by using the question “Do the following factors increase your strain at your work: draught, noise, poor indoor climate, heat, cold, blinding lighting and restlessness of work environment (noisy and restless workplace)” on a scale 1 (very little) to 5 (very much) (Lehto and Sutela 2009)?. In this study, we summed all seven items into a single variable ranging from 7 to 35. The variable was dichotomized into “low” (7–18) and “high” (19–35) using the median value as a cutoff point.

A modified version of the validated Nordic Musculoskeletal Questionnaire (Kuorinka et al. 1987) was used to assess musculoskeletal pain. The questions asked whether the employee had experienced pain, aching or numbness in four anatomical areas (hands or upper extremities; neck or shoulders; lower back; and feet or lower extremities) during the preceding week, with the reply scale being from 0 (not at all) to 10 (very much). The variables were dichotomized at the median (less than median: 0 = no or mild; more than median: 1 = severe). The cutoff values for upper extremities, neck and shoulder, lower back and lower extremities were 4, 5, 2 and 2, respectively, and the cutoff values were included in no or mild category. The dichotomized variables were then summed into a variable expressing the number of areas with severe pain (from zero to four) and further dichotomized into no (0 or 1) and yes (2–4) multisite pain (Neupane et al. 2018).

The age of the workers was used as continuous score, gender classified as female and male and the study group as senior program vs. controls.

### Statistical analysis

The baseline characteristics of the study participants are presented as frequencies and percentages by the study group and the differences were tested using a Chi-square test for the categorical exposure variables and ANOVA for the continuous variables. We used Cox proportional hazards analysis to estimate hazard ratios (HRs) with their 95% confidence intervals (CIs) for both the outcomes:; early exit from the labor market and the disability pension. We first checked the proportional hazard assumption for all exposure variables. The exact date of retirement was obtained from the Finnish pension register and used to calculate the follow-up time from the year of the survey the participant responded. Those employees who received a pension regardless of the type of pension prior to the baseline survey were excluded. Next, participation in the intervention program and selected work and non-work-related variables were studied as predictors of both the outcomes (early exit from labor market and disability pension) separately. First, we investigated the crude associations of the predictors with the outcomes (Model I). Second, Model II was a multivariable model simultaneously adjusted for all studied variables from the univariate models.

Nelson–Aalen cumulative hazard estimates (crude hazard) were plotted for both outcomes: early exit from labor market and disability pension by study group. Nelson–Aalen cumulative hazard measures the total amount of risk accumulated up to a certain time of follow-up and provides the cumulative number of expected events (early exit from labor market and disability pension in this case) within a certain period of time.

In a sensitivity analysis, the follow-up was calculated as the time from the year at which participants enrolled in the senior program, or for the controls to the date of retirement, to examine the distribution of individual risk time between the control and the intervention. The results are presented separately in a supplementary file. We also plotted Nelson–Aalen cumulative hazard estimates curve for both the outcomes using age as follow-up time to see the changes in crude hazard at different age by the study group.

All statistical analysis was conducted using Stata v17.

## Results

Overall, more women (73%) were included in the study compared to men. Similarly, in the senior program more women participated compared to the control group (85% vs. 65%). The participants mean age was 55.8 (SD 3.8) years. At baseline, both the intervention and controls were similar except for musculoskeletal pain (Table 1). No statistically significant difference was observed between the study groups for work ability, although comparatively more workers in the controls had good work ability than in the senior program (32.5% vs. 29%). No significant differences in the distribution of other exposure variables (repetitive movements, awkward postures, and environmental exposure) between the study group were found. Significantly more employees in the senior program had multisite pain (72.7% vs 57.6%).

**Table 1:** Characteristics of the study population by study group

Characteristics	Total *N* = 259	Study group		*P* value
		Senior program *n* = 107	Control *n* = 152	
Age, mean (SD)	55.76 (3.79)	55.31 (3.25)	56.08 (4.11)	0.101
Gender				< 0.001
Women	193 (72.83)	93 (84.55)	100 (64.52)	
Men	72 (27.17)	17 (15.45)	55 (35.48)	
Work ability				0.831
Poor (0–7)	81 (31.40)	35 (32.71)	46 (30.46)	
Moderate (8)	97 (37.60)	41 (38.32)	56 (37.09)	
Good (9–10)	80 (31.01)	31 (28.97)	49 (32.45)	
Physical strain				0.765
Low	121 (46.90)	49 (45.79)	72 (47.68)	
High	137 (53.10)	58 (54.21)	79 (52.32)	
Repetitive movements				0.884
Low	157 (60.15)	65 (59.63)	92 (60.53)	
High	104 (39.85)	44 (40.37)	60 (39.47)	
Awkward posture				0.579
Low	161 (62.16)	65 (60.19)	96 (63.58)	
High	98 (37.84)	43 (39.81)	55 (36.42)	
Environmental exposure				0.714
Low	84 (35.74)	36 (37.11)	48 (34.78)	
High	151 (64.26)	61 (62.89)	90 (65.22)	
Musculoskeletal pain				0.016
No	88 (36.21)	27 (27.27)	61 (42.36)	
Multisite pain	155 (63.79)	72 (72.73)	83 (57.64)	

The mean follow-up of time for those in the senior program was 7.42 years (95% CI 6.42–8.09), compared to the control 6.63 years (95% CI 6.25–7.52) (Table 2). Table 3 shows the potential predictors of disability retirement. In total, 51 employees (19.2%) exited early from the labor market (Table 3). A higher proportion of employees in the control group (23.7%) exited early compared to 14% in the senior program group. Similarly, early exit from labor market rate was 26% among men vs 17% among women. Almost one-third (30%) of those with poor work ability exited early, compared to 23% of those with moderate work ability and 4% of those with good work ability. In relation to early exit for the high and low work exposure group, almost no difference was found for physical strain, repetitive movements, awkward posture and environmental exposure. For those employees with multisite musculoskeletal pain, 24% exited from labor market early compared to 11% in those with no multisite pain.

**Table 2:** Number of disability pension and mean follow-up time by study group

	Early labor market exit	Mean follow-up time (years) and 95% CI
Senior program (*n* = 107)	15	7.42 (6.42–8.09)
Control group (*n* = 152)	36	6.63 (6.25–7.52)

**Table 3:** Predictors of early labor market exit. Hazard ratio (HR) and their 95% confidence intervals (CIs)

Characteristics	Early labor market exit		
	*n* = 51^a^ (%)	Model I	Model II
Study group			
Control	23.7	1	1
Senior program	14.0	0.56 (0.30–1.05)	0.40 (0.19–0.84)
Age		0.95 (0.88–1.02)	0.96 (0.89–1.03)
Gende**r**			
Female	17.4	1	1
Male	26.1	1.56 (0.86–2.82)	1.27 (0.60–2.67)
Work ability			
Poor	30.0	1	1
Moderate	23.2	0.61 (0.33–1.12)	0.57 (0.28–1.16)
Good	3.9	0.09 (0.03–0.30)	0.06 (0.01–0.29)
Physical strain			
Low	20.5	1	1
High	18.5	0.94 (0.52–1.68)	0.60 (0.29–1.23)
Repetitive movements			
Low	19.1	1	1
High	19.4	1.27 (0.70–2.29)	1.35 (0.60–3.04)
Awkward posture			
Good	18.6	1	1
Poor	20.6	1.31 (0.73–2.37)	1.07 (0.50–2.32)
Environmental exposure			
Low	19.8	1	1
High	20.1	1.27 (0.67–2.41)	0.76 (0.37–1.56)
Musculoskeletal pain			
No	11.4	1	1
Multisite pain	23.5	2.50 (1.19–5.23)	2.31 (0.92–5.79)

Model I: crude model. Model II: simultaneous adjustment of all variables included in the model

^a^Number of subjects with early labor market exit

Employees in the senior program had a 60% lower risk of early exit from labor market (HR 0.40, 95% CI 0.19–0.84) in the multivariable adjusted model (model II, Table 3) compared to the controls. The risk of early exit from labor market between men and women was not statistically significant. Having good work ability was strongly associated with a lower risk of early exit from labor market compared to poor work ability, in both the bivariate and multivariable models. The association was stronger in the multivariate model (HR 0.06, 95% CI 0.01–0.29).

Among other work-related variables, those exposed to high physical strain had a lower risk of early exit from labor market, although the association was not statistically significant. Those reporting being exposed to high repetitive movements had a 35% higher risk of early exit from labor market in the multivariable model, although the result was not statistically significant. Also, not statistically significant, poor awkward postures were associated with a 31% higher risk of early exit from labor market in a bivariate model, but the association was attenuated in the multivariable model. Those exposed to high environmental exposures had a 27% higher risk of early exit in the bivariate model; however, the association reversed in the multivariable model, with no statistical significance. Employees with multisite pain had 2.5-fold higher risk of early exit in the bivariate model (HR 2.5, 95% CI 1.19–5.23). However, the significant association was lost in the multivariable model.

**Fig. 1:**
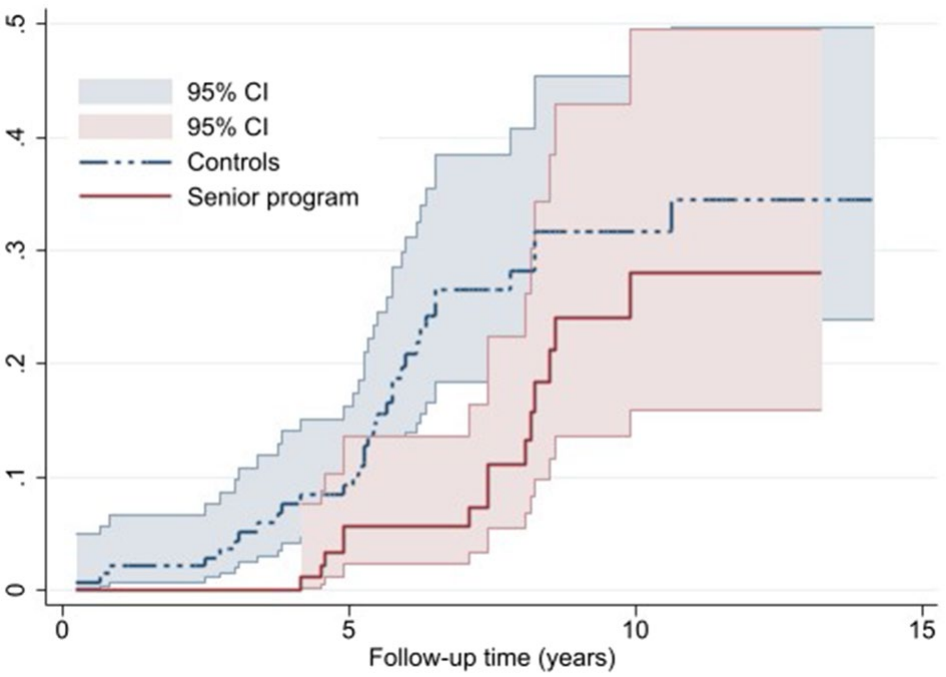
Nelson–Aalen cumulative hazard curve for early labor market exit by study group

Nelson–Aalen cumulative hazard estimates of early exit from labor market by the study group is shown in Fig. 1. The estimates in the senior program and controls started to diverge prior to the 5-year of follow-up. Differences in estimates became wider after 5 years. Cumulative hazard estimates were higher for the controls throughout the follow-up period.

Table 4 shows the predictors of disability retirement. Participants in the senior program had a 60% lower risk of disability retirement (HR 0.40, 95% CI 0.16–0.99). The magnitude of the effect remained the same in the multivariable model, but the significance of the association was lost. Males were at higher risk of disability retirement (HR 2.73, 95% CI 1.08–6.90) compared to females. The association was statistically significant in both the bivariate and multivariate models. Employees with good work ability had a lower risk of disability retirement compared to those with poor work ability both in the bivariate and multivariable models (HR from multivariable model 0.15, 95% CI 0.03–0.73). Multisite musculoskeletal pain was strongly associated with disability retirement (HR 4.08, 95% CI 1.09–15.34), which was statistically significant only in the multivariable model. Other work-related variables showed no significant association with disability retirement both in the bivariate and multivariate models.

**Table 4:** Predictors of disability retirement. Hazard ratio (HR) and their 95% confidence intervals (CIs)

Characteristics	Disability retirement (*n* = 27)	
	Model I	Model II
Study group		
Control	1	1
Senior program	0.40 (0.16–0.99)	0.40 (0.15–1.08)
Age	1.01 (0.99–1.04)	0.99 (0.90–1.09)
Gender		
Female	1	1
Male	1.55 (1.06–2.26)	2.73 (1.08–6.90)
Work ability		
Poor	1	1
Moderate	0.79 (0.51–1.23)	0.75 (0.31–1.84)
Good	0.42 (0.26–0.70)	0.15 (0.03–0.73)
Physical strain		
Low	1	1
High	1.02 (0.69–1.48)	1.25 (0.49–3.16)
Repetitive movements		
Low	1	1
High	0.95 (0.64–1.40)	1.01 (0.37–2.79)
Awkward posture		
Good	1	1
Poor	1.38 (0.94–2.02)	0.74 (0.27–2.02)
Environmental exposure		
Low	1	1
High	1.20 (0.81–1.76)	0.80 (0.32–2.05)
Musculoskeletal pain		
No	1	1
Multisite pain	1.46 (0.96–2.21)	4.08 (1.09–15.34)

Model I: crude model. Model II: simultaneous adjustment of all variables included in the model

**Fig. 2:**
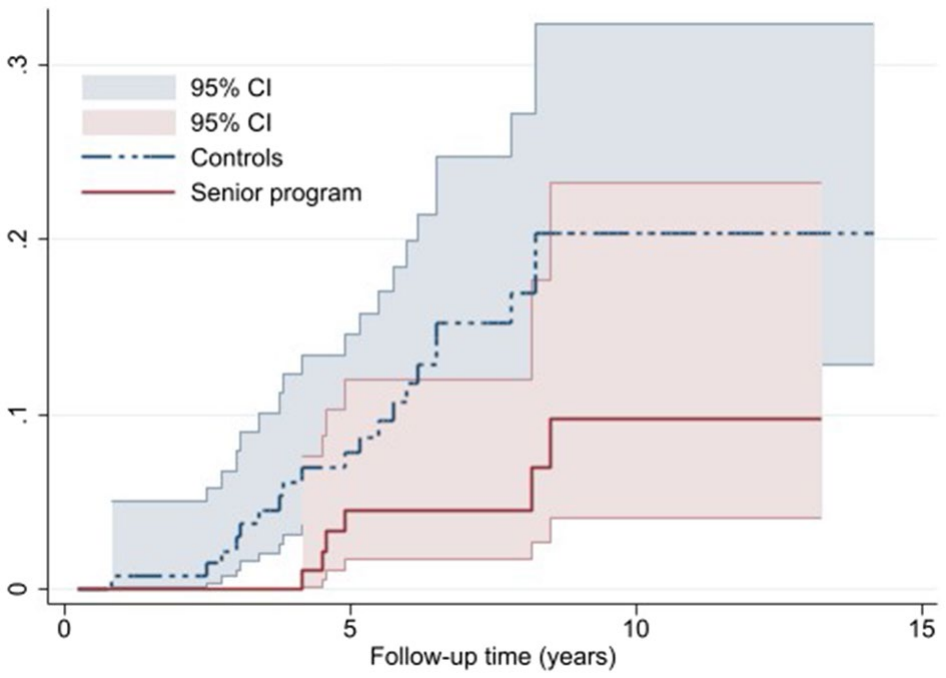
Nelson–Aalen cumulative hazard curve for disability pension by study group

Figure 2 shows the Nelson–Aalen cumulative hazard estimates of disability pension by the study group. The estimates show small difference in the first 1–2 years of the follow-up, after which divergence was observed. Differences in estimates became wider after 5 years and further increased after 10 years of follow-up.

Following sensitivity analysis, to examine the distribution of individual risk time between the study group, we calculated the follow-up time from the year when the participants were enrolled in the senior program, or in the controls to the date of retirement (event), and the results were a little different. The mean follow-up time was shorter for the intervention compared to the controls (Table S1). However, we found no major changes in the hazard estimates except that some of the estimates were statistically non-significant (Tables S1–S4 and Figs. S1–S4).

## Discussion

This study found that those in the senior program worked longer (7.42, 95% CI 6.42–8.09) compared to the control group (6.63, 95% CI 6.25–7.52). A workplace intervention was found to reduce the likelihood of early exit from labor market and also on the need to access a disability pension among older industrial workers. In relation to the work-related factors, only work ability was significantly associated with early exit from labor market. Employees with good work ability had a 94% reduced risk of exiting the labor market compared to those who reported poor work ability. In our analyses, physical working conditions were not significant predictors of whether an employee was likely to exit earlier than retirement age from the labor market. Moreover, being of male gender was significantly associated with accessing the disability pension. Having good work ability was associated with 95% lower risk of having disability pension and having multisite musculoskeletal pain was associated with higher risk of accessing disability pension.

The intervention program was initially implemented to reduce costs related to high rates of sickness absence and early retirement, mainly due to musculoskeletal disorders. The intervention program offered options to adjust individual work arrangements according to an employees’ work ability (Siukola 2013). The senior program was associated with lower risk for long sickness absence (Siukola et al. 2011). Furthermore, long sickness absence has been associated with increased risk of accessing a disability pension (Salonen et al. 2018; Kivimäki et al. 2004). We identified only a few studies which had investigated the effect of interventions to prevent early retirement and reduce the risk of requiring a disability pension. An earlier Dutch study reported an occupational health intervention program focused on at least three consultations with employee’s supervisor and an assessment interview prevented early retirement (Boer et al. 2004). The study found fewer early retirement in the intervention group compared to the control group at 2 years of follow-up (Boer et al. 2004). A Norwegian study identified lower rates of disability pension among employees with reduced work capacity in an intervention group that received work adjustments, including the use of technical aids, change of role or work tasks or reduced working hours, (Midtsundstad and Nielsen 2016). In line with our findings, a Finnish study reported that the affiliation of an occupational health care unit with the quality network as an intervention resulted in reduced numbers of employees requiring a full permanent or provisional disability pensions (Kuronen et al. 2020). The risk of work disability was also reduced among Finnish employees in a vocationally oriented multidisciplinary intervention program, which involved a learning process to support the maintenance of employees’ long-term work ability (Suoyrjö et al. 2009). Contrary to this, de Boer et al. (2007) reported no change in employees accessing a disability pension who were in an intervention group in 18 and 26 months of follow-up.

The content of the interventions in earlier studies varied: some studies changed working conditions such as work adjustments, a change of the role or work task, reduced working hours (Midtsundstad and Nielsen 2016; Boer et al. 2004), being affiliated with a quality network of occupational healthcare (Kuronen et al. 2020), and counselling and education program (Boer et al. 2007). In our study, the intervention included the options of choices relating to adjusting work arrangements in relation to an employee’s individual work ability (Siukola 2013), which has similarities to an earlier Dutch (Boer et al. 2004) and Norwegian (Midtsundstad and Nielsen 2016) study in some respects. However, we do not have information on which components of the intervention had the desired impact in our study.

We also studied which work-related factors were associated with early exit from labor market and disability retirement. Our results show that work ability was a significant predictor of early exit from the labor market and also for the disability pension. Employees with good work ability had 94% lower risk of early exit from labor market and 85% lower risk of disability pension compared to employees with poor work ability. Similar findings have been reported in earlier observational studies, while many intervention studies have studied work ability as an outcome rather than as a predictor of early retirement. Earlier studies from Finland (Jääskeläinen et al. 2016) and the Netherlands (Roelen et al. 2014) demonstrated that poor work ability was associated with work disability pension.

We found no statistically significant association of other physical working conditions with early exit and disability pension, although higher risk of disability was found among those who reported awkward postures or repetitive movements at work for early exit from the labor market. These findings are in line with earlier studies that have shown strong associations of physical workload with disability retirement among municipal employees (Lahelma et al. 2012; Halonen et al. 2020). Higher levels of physical exertion or poor physical ergonomic exposures were associated with being on a disability pension among older workers in Denmark (Andersen et al. 2020; Labriola et al. 2009). A potential explanation is the crude categorization of the exposure variables, which might have failed to discriminate between actual exposure levels in our study. Moreover, having multisite pain was associated with higher risk of early exit from labor market and also for disability pension, but after adjusting for work ability and other work-related factors, the association was attenuated for the early exit from labor market. The proportion of employees exiting from the labor market was 24%among those with multisite pain, while it was 11% among the group with no multisite pain. Multisite musculoskeletal pain has previously been reported as a strong predictor of disability pension (Haukka et al. 2015; Hiilamo et al. 2021).

We found no difference in risk of early exit from labor market by gender or age, although more men took early exit compared to women (26% vs. 17%). However, we found a significant association of males with disability retirement even following adjustment for work-related factors, which supports findings from a Norwegian study that reported a lower risk of disability pension among women and in older employees (Midtsundstad and Nielsen 2016). The contrasting results of the differences in male and female for early exit from labor market in our study are likely explained by the relatively small sample size of the current study and the distribution of gender between the two study groups.

In an additional analysis when the follow-up time was calculated from the year when the participants were enrolled in the senior program or in the controls to the date of retirement (event), the estimates turned out to be insignificant. Also, the mean follow-up time was shorter for the intervention compared to the controls.

A limitation of the current study is the small sample size, which may reduce the potential for statistically significant results. A small sample was due to the intervention being “a real-life experiment” as opposed to a controlled trial with power calculations. For similar reasons, adjustment to the final model with all potential confounders, such as morbidity, was also limited. Only a few cases (*n* = 27) were accessing a full-time disability pension. Although larger sample sizes and more events are always preferable, situations commonly arise where confounding cannot be persuasively addressed without violating the rule of thumb (Vittinghoff and McCulloch 2007). In this case, the results should be interpreted with caution.

A key strength of the current study is the long follow-up of an age management program, which was nationally and internationally awarded as a best practice workplace program. The study included all workers in the company aged 55 years or over, either in the intervention or the control group (Siukola et al. 2011). Participation in the intervention program was voluntary, as such no randomization was possible. A randomization in the workplace may have been ethically questionable. The baseline measurement of the exposure was used from different surveys from 2003 to 2009 and the employees involved in the intervention at different times from 2004 onward; however, information on the number of years that employees had participated in the intervention program was not available. It is possible that the limited knowledge on years of participation may have impacted our findings; however, identical survey questions were used at each time point and the content of the intervention was also consistent. The intervention program was later offered as a routine workplace health program through occupational healthcare for all workers. Some of the control group may then have participated in the program after the intervention, resulting in potential dilution of our estimate of the effect of the intervention on the outcomes.

In conclusion, this study shows that the nationally and internationally awarded age management ‘senior program’ had a strong positive impact on both the early labor market exit and prolonging work life and reducing disability pensions among the industrial employees. Good work ability was associated with lower risk and multisite musculoskeletal pain with higher risk of early labor market exit and disability pension. Policymakers may consider implementing age management programs for older employees to prolong work life and to reduce the risk of early exit from labor market and a disability pension. Promotion of strategies to maintain work ability and prevention of musculoskeletal pain should also be considered in workplace programs to facilitate sustainable employment opportunities and reduce the likelihood of employees exiting from labour market and requiring a disability pension. Increased numbers of aging workers and the need to stay at work for longer warrant larger-scale studies to expand on the current findings and inform policy relating to the design of workplace interventions aimed at reducing the risk of early exit and requiring a disability pension.

## Supplementary Information

Below is the link to the electronic supplementary material.

Supplementary file1 (DOCX 41 kb)

## Data Availability

Survey data can be shared with a request to the corresponding author. Data sharing requires an application stating the detailed purpose of data use. A data sharing agreement is required, and this must fulfill the requirements of the EU General Data Protection Regulation 2016/679 (GDPR).
