# Supplementary material for: Can workplace intervention prolong work life of older workers? A quasi-experimental study
Source: Int Arch Occup Environ Health. 2022 Sep 6;96(2):237–46. doi: 10.1007/s00420-022-01919-8 (PMC9905162; doi:10.1007/s00420-022-01919-8)
Supplement: Supplementary file 1 — Supplementary file1 (DOCX 41 kb) [file 420_2022_1919_MOESM1_ESM.docx]

**Supplementary results**

**Table S1:** Predictors of early labor market exit. Hazard ratio (HR) and their 95% confidence intervals (CIs).

| **Characteristics** | **Early labor market exit** | | |
| --- | --- | --- | --- |
|  | **n=51**^†^ Cases, % | **Model I** | **Model II** |
| **Study group**^†^ |  |  |  |
| Control | 23.7 | 1 | 1 |
| Senior program | 14.0 | 0.69 (0.37-1.30) | 0.58 (0.27-1.24) |
| **Age** |  | 0.93 (0.88-0.99) | 0.95 (0.89-1.00) |
| **Gender** |  |  |  |
| Female | 17.4 | 1 | 1 |
| Male | 26.1 | 1.44 (0.77-2.68) | 1.26 (0.56-2.81) |
| **Work ability** |  |  |  |
| Poor | 30.0 | 1 | 1 |
| Moderate | 23.2 | 0.77 (0.41-1.45) | 0.73 (0.35-1.53) |
| Good | 3.9 | 0.11 (0.03-0.39) | 0.09 (0.02-0.42) |
| **Physical strain** |  |  |  |
| Low | 20.5 | 1 | 1 |
| High | 18.5 | 0.91 (0.50-1.67) | 0.61 (0.28-1.32) |
| **Repetitive movements** |  |  |  |
| Low | 19.1 | 1 | 1 |
| High | 19.4 | 1.16 (0.63-2.15) | 1.16 (0.46-2.92) |
| **Awkward posture** |  |  |  |
| Good | 18.6 | 1 | 1 |
| Poor | 20.6 | 1.35 (0.73-2.48) | 1.35 (0.55-3.31) |
| **Environmental exposure** |  |  |  |
| Low | 19.8 | 1 | 1 |
| High | 20.1 | 1.39 (0.70-2.75) | 0.89 (0.41-1.95) |
| **Musculoskeletal pain** |  |  |  |
| No | 11.4 | 1 | 1 |
| Multisite pain | 23.5 | 2.25 (1.06-4.76) | 2.00 (0.78-5.12) |

^†^ Mean follow-up time 5.55 years (95% CI 5.11-5.98) for control group, and 5.11 years (95% CI 4.64-5.57) for senior program group.

Model I: crude model

Model II: simultaneous adjustment of all variables included in the model

**Table S2:** Predictors of disability retirement. Hazard ratio (HR) and their 95% confidence intervals (CIs).

| **Characteristics** | **Disability retirement (n=27)** | |
| --- | --- | --- |
|  | **Model I** | **Model II** |
| **Study group** |  |  |
| Control | 1 | 1 |
| Senior program | 0.46 (0.18-1.16) | 0.49 (0.18-1.34) |
| **Age** | 0.94 (0.86-1.02) | 0.97 (0.90-1.05) |
| **Gender** |  |  |
| Female | 1 | 1 |
| Male | 3.03 (1.38-2.65) | 2.66 (1.00-7.09) |
| **Work ability** |  |  |
| Poor | 1 | 1 |
| Moderate | 0.74 (0.31-1.74) | 0.85 (0.34-2.15) |
| Good | 0.14 (0.03-0.65) | 0.17 (0.03-0.86) |
| **Physical strain** |  |  |
| Low | 1 | 1 |
| High | 1.18 (0.52-2.69) | 1.30 (0.48-3.47) |
| **Repetitive movements** |  |  |
| Low | 1 | 1 |
| High | 0.90 (0.38-2.11) | 0.87 (0.28-2.73) |
| **Awkward posture** |  |  |
| Good | 1 | 1 |
| Poor | 1.35 (0.59-3.08) | 0.92 (0.29-2.86) |
| **Environmental exposure** |  |  |
| Low | 1 | 1 |
| High | 1.69 (0.67-4.30) | 0.88 (0.31-2.48) |
| **Musculoskeletal pain** |  |  |
| No | 1 | 1 |
| Multisite pain | 4.36 (1.29-14.74) | 3.81 (1.00-14.57) |

Model I: crude model

Model II: simultaneous adjustment of all variables included in the model

**Table S3:** Participation in the questionnaire survey by the survey year

|  | **Survey year** | | | |
| --- | --- | --- | --- | --- |
|  | **2003** | **2005** | **2007** | **2009** |
| **Senior program** | 70 (65.4) | 16 (15.0) | 18 (16.8) | 3 (2.8) |
| **Controls** | 49 (32.2) | 63 (41.5) | 26 (17.1) | 14 (9.2) |
| **Total (n=259)** | 119 | 79 | 44 | 17 |

**Table S4:** Participants joined one of the study groups by year

| **Study group** | **Started year** | | | | | |
| --- | --- | --- | --- | --- | --- | --- |
|  | **2004** | **2005** | **2006** | **2007** | **2008** | **Total** |
| **Senior program** | 30 (28.1) | 15 (14.0) | 23 (21.5) | 24 (22.4) | 15 (14.0) | 107 |
| **Controls** | 78 (51.3) | 14 (9.2) | 21 (13.8) | 21 (13.8) | 18 (11.9) | 152 |

**Figure S1:** Nelson-Aalen cumulative hazard curve for early labor market exit by study group. Follow-up time was calculated from the year when participants joined one of the study groups to the date of retirement

**Figure S2:** Nelson-Aalen cumulative hazard curve for disability pension by study group. Follow-up time was calculated from the year when participants joined one of the study groups to the date of retirement

**Figure S3:** Nelson-Aalen cumulative hazard curve for early labour market exit by study group. Age was used as a follow-up time.

**Figure S4:** Nelson-Aalen cumulative hazard curve for disability pension by study group. Age was used as a follow-up time.
